# Comments to the “Letter to the Editor” for the manuscript titled “Clinico‐sero‐pathological characteristics of anti‐Ha antisynthetase syndrome”

**DOI:** 10.1111/bpa.70014

**Published:** 2025-05-14

**Authors:** Bing Zhao, Lining Zhang, Tingjun Dai

**Affiliations:** ^1^ Department of Neurology, Qilu Hospital (Qingdao), Cheeloo College of Medicine Shandong University Qingdao China; ^2^ Department of Neurology Research Institute of Neuromuscular and Neurodegenerative Diseases, Qilu Hospital of Shandong University, Shandong Provincial Key Laboratory of Mitochondrial Medicine and Rare Diseases Jinan China; ^3^ Department of Rheumatology, Shandong Key Laboratory of Medicine and Prevention Integration in Rheumatism and Immunity Disease Qilu Hospital of Shandong University Jinan China

Thank you very much for presenting this instructive case, which demonstrates classical features of anti‐synthetase syndrome (ASS) through its clinical triad (arthritis, myositis, and interstitial lung disease) and characteristic muscle pathology (perifascicular necrosis with MHC‐II overexpression). We would like to provide the following insights based on our experience:

## 1 ANTIBODY DETECTION METHODOLOGIES

In our previous studies, when anti‐Ha antibodies were identified via immunoblotting, we further validated results using cell‐based assays (CBA) and immunoprecipitation (IP). However, significant discrepancies were observed across these methods. For instance, some samples showed strong positivity in IP but weak reactivity in CBA, while others exhibited faint IP signals despite prominent immunoblot bands. We hypothesize that these inconsistencies may arise from: (1) variations in the conformational structure of the Ha‐antigen complex across different platforms; (2) technical differences in antigen presentation and assay conditions. While IP remains the current gold standard, our findings highlight the need for harmonized protocols to improve cross‐method reproducibility.

## 2 HETEROGENEITY IN CLINICAL AND PATHOLOGICAL PRESENTATIONS

Although the classic ASS triad and perifascicular necrosis are well recognized, our cohort of anti‐Ha‐positive patients rarely presented with such prototypical features as described in this report by Marie‐Thérèse Holzer et al. Potential explanations for this divergence include: (1) Selection bias: there might be some differences in patient referral patterns between neuromuscular centers (focused on myopathy subtypes) and rheumatology centers (prioritizing systemic manifestations); (2) Genetic predispositions: Population‐specific HLA haplotypes or modifier genes may influence phenotypic expression.

We aim to publish our previous findings to raise awareness of anti‐Ha‐associated ASS heterogeneity and encourage multicenter collaborative efforts to validate these observations across diverse populations. Enhanced recognition of anti‐Ha antibody cases through interdisciplinary collaboration will ultimately refine the clinicopathological profile of this rare ASS subtype and optimize therapeutic strategies.

## AUTHOR CONTRIBUTIONS

**Bing Zhao**: Writing—Original draft preparation. **Lining Zhang, Tingjun Dai**: Writing—Reviewing and Editing.

## Data Availability

Research data are not shared.

